# The exon 38-containing ARHGEF11 splice isoform is differentially expressed and is required for migration and growth in invasive breast cancer cells

**DOI:** 10.18632/oncotarget.20985

**Published:** 2017-09-18

**Authors:** Masahiko Itoh, Derek C. Radisky, Masaaki Hashiguchi, Hiroyuki Sugimoto

**Affiliations:** ^1^ Department of Biochemistry, School of Medicine, Dokkyo Medical University, Mibu, Tochigi, Japan; ^2^ Department of Cancer Biology, Mayo Clinic, Jacksonville, Florida, USA; ^3^ Department of Immunology, School of Medicine, Dokkyo Medical University, Mibu, Tochigi, Japan

**Keywords:** breast cancer, epithelial-mesenchymal transition, invasion, alternative splicing, RhoGEF

## Abstract

Breast cancer invasion involves the loss of cell-cell junctions and acquisition of an invasive, migratory phenotype, and breast cancer cells of the basal intrinsic subtype are more invasive and metastatic than breast cancer cells of other subtypes. ARHGEF11 is a RhoGEF that was previously shown to bind to the tight junction protein ZO-1 at perijunctional actomyosin ring (PJAR), a network of cortically organized actin and myosin filaments associated with junctional complexes that regulates cell-cell adhesion and polarization. We show here that ARHGEF11 shows splice isoform expression that differs according to the intrinsic subtype of breast cancer cells and that controls their invasive phenotype. Luminal subtype breast cancer cells express the isoform of ARHGEF11 lacking exon 38 (38-), which binds to ZO-1 at PJAR and is necessary for formation and maintenance of cell-cell junctions. Basal subtype breast cancer cells express the isoform of ARHGEF11 containing exon 38 (38+), which does not bind to ZO-1 and which drives cell migration and motility. Depletion of ARHGEF11 in basal subtype breast cancer cells is sufficient to alter cell morphology from a mesenchymal stellate form with extensive cell protrusions to a cobblestone-like epithelial form, and to suppress growth and survival both *in vitro* and *in vivo*. These findings show that the expression of the particular splice isoform of ARHGEF11 is critically linked to the malignant phenotype of breast cancer cells, identifying ARHGEF11 exon 38(+) as a biomarker and target for therapy of breast cancer.

## INTRODUCTION

Disruption of the organized epithelial architecture is an essential requirement of tumor invasion and metastasis. Adherens junctions (AJ) and tight junctions (TJ) are sites of intercellular adhesion that maintain the integrity of epithelial tissues and regulate intracellular signaling and bind to the cortical actin cytoskeleton at the perijunctional actomyosin ring (PJAR) [1]. AJ provides selective and strong mechanical connection between adjacent epithelial cells, and TJ are required for establishment of cell polarity. The cytoplasmic components ZO-1 and ZO-2 recruit TJ proteins at the most apical part of epithelial cells to anchor to the PJAR [2, 3]. Previously, we identified ARHGEF11 as a RhoGEF that regulates PJAR [4]. ARHGEF11 is localized at TJ by a direct interaction with ZO-1, and depletion of ARHGEF11 inhibits the formation of TJ and PJAR in nonmalignant mammary epithelial cells [5]. However, how regulation of ARHGEF11 could affect the invasive phenotype of breast cancer cells was unknown.

Dysregulation of AJ and TJ is necessary for invasion and metastasis of epithelial-derived cancer cells. The most well-known mechanism associated with coordinated loss of AJ and TJ in association with the cancer progression is epithelial-mesenchymal transition (EMT) [6]. During EMT, cortical organization of PJAR is lost, AJ as well as TJ are disrupted, and epithelial cells are transformed into mesenchymal, migratory cells with an increased mobility into surrounding environment. Previous studies have demonstrated that activation of key transcriptional regulators such as Snail and Twist can induce EMT during cancer progression [7], and one key mechanism by which these regulators accomplish this is through activation of alternative splicing programs that drive EMT [8]. Recent studies have shown that activation of EMT by expression of Twist in human mammary epithelial cells is associated with a broad array of splice isoform alterations, including increased expression of ARHGEF11 isoform [9].

Here we analyzed the functional consequences of ARHGEF11 splice isoform expression in breast cancer cells. We identified striking and consistent differences in the particular splice isoforms expressed in noninvasive luminal subtype breast cancer cell lines vs invasive basal subtype breast cancer cell lines. We found that the alternative splice isoform expressed in basal subtype breast cancer cells functions very differently from the previously identified functions of the isoform found in normal mammary epithelial cells and in luminal subtype breast cancer cells, and moreover that depletion of the ARHGEF11 isoform expressed in invasive breast cancer cells suppressed their malignant phenotype both *in vitro* and *in vivo*. Our study provides clear evidence that the isoform expression of ARHGEF11 has particular functional relevance to the malignant phenotype of breast cancer cells, and identifies ARHGEF11 as a potential target for cancer therapy.

## RESULTS

### The ARHGEF11 splice isoform expressed in invasive breast cancer cells lacks ZO-1- binding ability and targeting to cell-cell junctions

**Figure 1:**
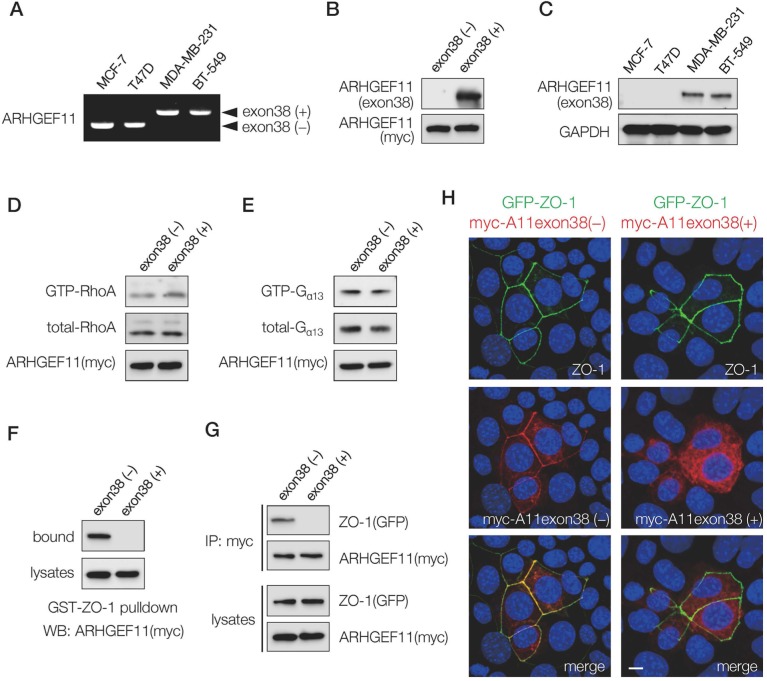
Expression and characterization of ARHGEF11 isoform in breast cancer cells **(A)** RT-PCR analysis with primers flanking the exon 38 of ARHGEF11 in non-invasive (MCF-7, T47D) and invasive (MDA-MB-231, BT-539) breast cancer cells. **(B)** The cell lysates from 293T cells transfected with myc-tagged ARHGEF11exon38(-) or ARHGEF11exon38(+) were processed for western blotting with the antibody against amino acids coded by exon 38 or myc. **(C)** The western blot analysis of non-invasive (MCF-7, T47D) and invasive (MDA-MB-231, BT-539) breast cancer cells with anti-ARHGEF11exon38(+) specific antibody. **(D, E)** GTP-bound active RhoA (D) or GTP-bound active G_α13_ (E) was pulled-down from the lysates of 293T cells transfected with myc-tagged ARHGEF11exon38(-) or ARHGEF11exon38(+) using GST-Rhotekin RBD or GST-TPR, respectively. RhoA or G_α13_ in bound fractions or total cell lysates were detected by western blotting. **(F, G)** The interaction between ZO-1 and ARHGEF11exon38(-) or ARHGEF11exon 38(+) was assessed by pull-down assay using GST-ZO-1 (F) or immunoprecipitation (G). **(H)** The localization of ZO-1 and ARHGEF11exon38(-) or ARHGEF11exon38(+) was analyzed by immunofluorescence. The mammary epithelial cells knockout for ZO-1 were co-transfected with GFP-tagged ZO-1 and myc-tagged ARHGEF11exon38(-) or ARHGEF11exon 38(+), then processed for immunostaining. Scale bar, 3μm.

Previous investigations suggested that induction of invasive mesenchymal characteristics in human mammary epithelial cells is associated with a splice isoform switch of ARHGEF11, from an isoform that lacks exon 38 that is expressed in normal mammary epithelial cells towards an isoform that expresses exon 38 in invasive variants [9]. We assessed expression of these isoforms in noninvasive luminal subtype breast cancer cell lines MCF-7 and T47D, and in invasive basal subtype breast cancer cell lines MDA-MB-231 and BT-549, and found that the luminal subtype cells expressed only the isoform lacking exon 38, henceforth referred to as A11exon38(-), and that the basal subtype cells expressed only the isoform containing exon 38, henceforth referred to as A11exon38(+) (Figure 1A).

To confirm that the protein expression of the A11exon38(+) isoform followed the pattern observed by RNA assessment, we generated an antibody recognizing the specific amino acids coded by exon 38. The antibody reacted with the exogenously expressed A11exon38(+) but not A11exon38(-) by western blots (Figure 1B). Using this antibody, we confirmed that A11exon38(+) protein was expressed in invasive breast cancer cells, while it was undetectable in non-invasive breast cancer cells (Figure 1C).

We then attempted to clarify molecular differences between A11exon38(-) and A11exon38(+) isoforms. Since the originally identified function of ARHGEF11 is as a RhoGEF [10], we compared Rho-activation ability of these isoforms by pull-down assay. Previous studies have shown that 293T cells do not have sufficient endogenous Rho-GTP to be measured using Rhotekin-GBD pull-down assays, while overexpression of ARHGEF11 increases the amount of GTP-bound RhoA [11]. 293T cells were transfected with myc-A11exon38(-) or myc-A11exon38(+) and active GTP-RhoA molecule was pulled-down by GST-Rhotekin RBD. As a result, almost same amount of active GTP-RhoA was detected in both samples (Figure 1D). ARHGEF11 is also known to promote GTP hydrolysis of Gα_13_ [12]. We tested A11exon38(-) and A11exon38(+) on Gα_13_ activity by pull-down using GST-TPR [13], and found similar amounts of GTP-Gα_13_ were detected in the cells transfected with either myc-A11exon38(-) or myc-A11exon38(+) (Figure 1E). These results indicated that the presence or absence of exon38 did not affect the function of ARHGEF11 for the regulation of Rho and Gα_13_.

In our previous study [5], we identified ARHGEF11 as a ZO-1-binding molecule by yeast two-hybrid screening. The isolated prey clones in that study encoded partial fragments of A11exon38(-), and this was the region of the molecule that we identified as having ZO-1 binding activity. We thus wondered whether inclusion of exon 38 affected the ability of ARHGEF11 to interact with ZO-1. First, we examined the interaction between ZO-1 and each ARHGEF11 isoform by pull-down assay. An expression vector containing myc-A11exon38(-) or myc-A11exon38(+) was introduced into 293T cells, and the cell lysates were incubated with GST-ZO-1 (1520-1745) which encoded ARHGEF11 binding domain in ZO-1. Western blots of the fractions bound to GST-ZO-1 (1520-1745) with an anti-myc antibody revealed the significant amounts of A11exon38(-) interacted with ZO-1, however, A11exon38(+) exhibited no observable interaction with GST-ZO-1 (1520-1745) (Figure 1F). The association of A11exon38(-) or A11exon38(+) with ZO-1 was also analyzed by immunoprecipitation (Figure 1G). The myc-A11exon38(-) or myc-A11exon38(+) was co-transfected with GFP-ZO-1 into 293T cells and the immunoprecipitation was performed using anti-GFP antibody. We found that A11exon38(-) efficiently co-precipitated with ZO-1, whereas A11exon38(+) did not.

We also assessed the ability of the different ARHGEF11 isoforms to co-localize with ZO-1 by immunofluorescence analysis. The ZO-1 knockout mouse mammary epithelial cell line, EpH4- ZO1-KO, was co-transfected with GFP-ZO-1 and myc-A11exon38(-) or myc-A11exon38(+) (Figure 1H). While myc-A11exon38(-) was co-localized with GFP-ZO-1 at cell-cell junctions, myc-A11exon38(+) was distributed throughout the cytoplasm. These results indicated that an important molecular difference between A11exon38(-) and A11exon38(+) isoforms is the differential ZO-1 binding ability and resultant subcellular localization. Therefore, A11exon38(+) isoform expressed in invasive basal subtype breast cancer cells probably plays a distinct role from ZO-1-dependent PJAR regulation identified for A11exon38(-).

### The A11exon38 (+) isoform regulates invasive behavior of MDA-MB-231 cells

**Figure 2:**
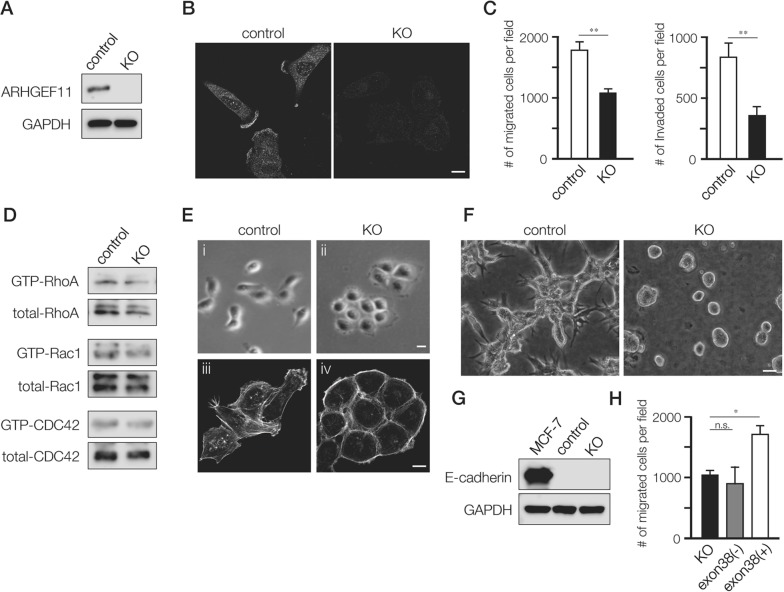
Generation and characterization of A11exon38(+) knockout MDA-MB-231 cells **(A)** The control MDA-MB-231 cells (control) and the cells knockout for A11exon38 (+) by CRISPR/Cas9 (KO) were analyzed by western blots with anti-ARHGEF11. **(B)** Immunofluorescence analysis of A11exon38 (+) in control and KO cells. Scale bar, 3μm. **(C)** The migration and invasion of control and KO cells were analyzed using transwell and boyden chamber, respectively. Data are mean±s.e.m. (n=3) ^**^P<0.01, unpaired two-tailed t-test. **(D)** The activity of RhoA, Rac1 and CDC 42 in control and KO cells were examined by pull-down assays. The GST-Rhotekin-RBD was used for detecting active-RhoA and GST-PAK-CRIB was used for detecting active-Rac1 and CDC42. **(E)** Phase-contrast images (i and ii) and actin filament staining (iii and iv) of control and KO cells. Scale bars, 3μm. **(F)** The morphology of control and KO cells cultured on top of matrigel. Scale bar, 15μm. **(G)** The expression of E-cadherin in MCF-7, control MDA-MB-231 and KO cells was determined by western blotting with anti-E-cadherin antibody. **(H)** The migration assay of KO cells, KO cells transfected with myc-A11exon38(-) or with myc-A11exon38(+) cells. Data are mean±s.e.m. (n=3) ^*^P<0.05, unpaired two-tailed t-test.

To define the role of A11exon38(+), we depleted ARHGEF11 in MDA-MB-231 cells using a CRISPR/Cas9-based approach. The MDA-MB-231 cells depleted of ARHGEF11, designated KO cells, were screened by western blots and confirmed by DNA sequence (Figure 2A). By immunofluorescence analysis, A11exon38(+) was detected at cell edge/fillopodia and cytoplasm in control MDA-MB-231 cells, while those signals were lost in KO cells (Figure 2B). We observed that KO cells appeared to have decreased motility in culture (data not shown); we quantified this phenotype using transwell migration assay and boyden chamber assays, finding that the KO cells exhibited significant reduction of both migration and invasion compared to control cells (Figure 2C). We assessed the effect of ARHGEF11 depletion in MDA-MB-231 cell on activity of Rho family molecules, and found that the level of GTP-bound active RhoA only slightly reduced in KO cells compared to control cells, and that active Rac1 and CDC42 levels were comparable between control and KO cells (Figure 2D).

In addition to decreased migration and invasion, KO cells exhibited altered cellular morphology. While control MDA-MB-231 cells showed the characteristic fibroblastic and scattered morphology of this cell type, KO cells exhibited rounded morphology and formed clusters like epithelial cells (Figure 2E, i and ii). In KO cells, the stress fiber formation was decreased and actin filaments showed a cortical-like organization (Figure 2E, iii and iv). The morphological difference was even more striking in cells cultured in 3D, where control cells were thin and elongated with prominent cellular protrusions, while KO cells formed tightly packed cell colonies lacking protrusions (Figure 2F).

While MDA-MB-231 cells do not normally express detectable E-cadherin, this molecule can be induced under certain conditions, such as the deletion of β1-integrin [14]. We wondered whether epithelial-like morphology of KO cells was due to the up-regulation of E-cadherin, but E-cadherin was not detected by western blotting (Figure 2G), indicating that epithelial-like characteristics of KO cells was independent of E-cadherin expression.

We also performed rescue experiments in which we introduced myc-A11exon38(-) or myc-A11exon38(+) expression vector into KO cells. Because we were unable to obtain stable transfectants, we utilized transient transfectants for migration and invasion assays. We found that expression of myc-A11exon38(+), but not myc-A11exon38(-), stimulated migration (Figure 2H) and invasion (data not shown). Taken together, these results show that A11exon38 (+) isoform is essential for the invasive phenotype of MDA-MB-231 cells.

### Expression of E-cadherin in MDA-MB-231 cells altered migration and invasion without affecting ARHGEF11 isoform switch

**Figure 3:**
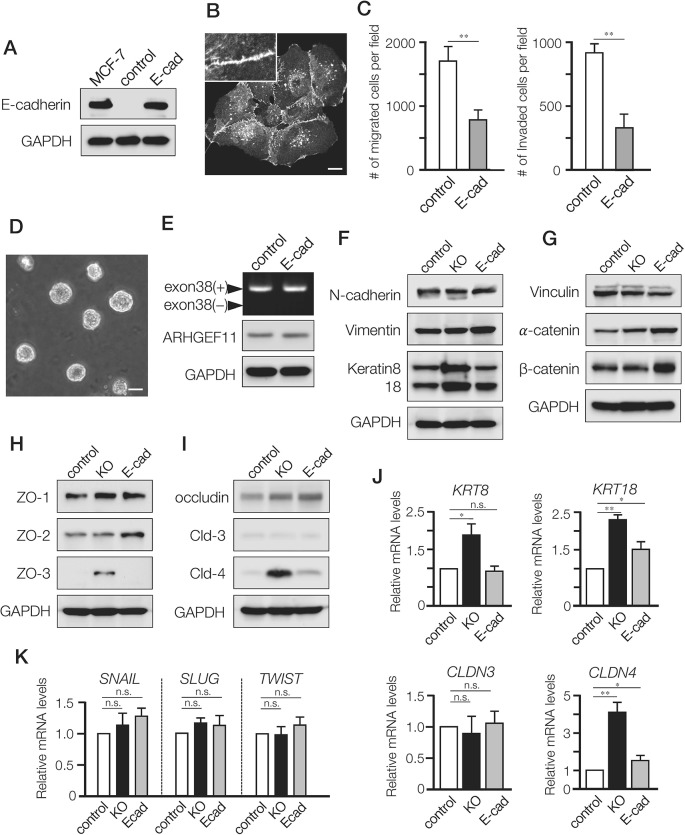
Differential effects of A11exon38(+) depletion and exogenous expression of E-cadherin in MDA-MB-231 cells **(A)** The expression of E-cadherin in MCF-7, control MDA-MB-231 cells (control) and MDA-MB-231 cells stably expressing exogenous E-cadherin (E-cad). **(B)** Immunofluorescence image of E-cadherin in the stable transfectants. Inset, higher magnification image. Scale bar, 3μm. **(C)** The migration and invasion of control and E-cad cells were examined using transwell and boyden chamber, respectively. Data are mean±s.e.m. (n=3) ^**^P<0.01, unpaired two-tailed t-test. **(D)** Phase contrast images of E-cad cell clusters cultured on top of matrigel. Scale bar, 15μm. **(E)** RT-PCR analysis with primers flanking the exon 38 of ARHGEF11 in control and E-cad cells (top panel). The protein expression of ARHGEF11 was determined by western blotting with anti-ARHGEF11 antibody (middle panel). **(F-I)** The western blotting analysis of mesenchymal markers (N-cadherin and vimentin), and epithelial markers keratin 8/18 (F), adherens junction components; vinculin, α-catenin, β-catenin (G), tight junction components; ZO-1, ZO-2, ZO-3 (H), membrane proteins of tight junctions; occludin, claudin-3 (Cld-3), claudin-4 (Cld-4) (I). **(J)** The relative mRNA levels of keratin8 (*KRT8*), keratin18 (*KRT18*), claudin-3 (*CLDN3*) and claudin-4 (CLDN4) were quantified by qRT-PCR analysis. Data are mean±s.e.m. (n=3) ^*^P<0.05, ^**^P<0.01, unpaired two-tailed t-test. (K) The relative mRNA levels of *SNAIL*, *SLUG* and *TWIST* in control, KO and E-cad cells were quantified by qRT-PCR analysis. Data are mean±s.e.m. (n=3) unpaired two-tailed t-test.

Our previous studies have shown how A11exon38(-) splice isoform expressed in noninvasive mammary epithelial cells is required for functional cell-cell junctions [5], and our current results suggested that the A11exon38(+) splice isoform expressed in invasive breast cancer cell lines both disrupts cell-cell junctions and promotes cell migration and motility. To dissect the differential effects of A11exon38(+) splice isoform on cell-cell junctions vs migration/motility, we generated MDA-MB-231 cells that exogenously express E-cadherin (designated E-cad cells, Figure 3A and 3B), which has been shown to inhibit invasiveness through regeneration of cell-cell junctions [15]. Similar to KO cells, E-cad cells exhibited reduced migration and invasiveness compared to control cells (Figure 3C), and when E-cad cells were cultured on top of matrigel, the cells formed spherical structures similar to KO cells (Figure 3D).

To test whether the introduced E-cadherin affected the isoform expression of ARHGEF11, we examined ARHGEF11 expression in E-cad cells by PCR analysis. We found that E-cad cells still expressed A11exon38(+) isoform (Figure 3E, top panel), and comparable amount of A11exon38(+) protein was detected in E-cad and control cells (Figure 3E, middle panel), indicating that expression of E-cadherin, which has been reported to induce some epithelial characteristics when exogenously expressed [15], is not sufficient to affect alternative splicing of ARHGEF11.

We then investigated the expressions of epithelial or mesenchymal molecules in the cell models. Compared with control cells, KO and E-cad cells exhibited comparable expressions of mesenchymal markers such as N-cadherin and vimentin (Figure 3F), but only KO cells showed substantially increased expression of keratin 8/18 (Figure 3F and 3J), which are markers of luminal mammary epithelial cells and utilized as epithelial markers [16]. We also assessed expression of apical junctional complex components in these cell lines, observing that while vinculin showed similar expression in all three lines, α- and β-catenin, which form complexes with E-cadherin, were elevated in E-cad cells compared to control (Figure 3G). That catenin expression was also unaltered in KO cells, provided further support for the concept that the role of A11exon38(+) in cell motility is independent of adherens junctions.

We observed strikingly different results when examining expression of tight junction components in these cell lines: while ZO-1, ZO-2 and occludin exhibited comparable expressions in all three cell lines, ZO-3, which exhibits specific expression in polarized epithelial cells *in vivo* and *in vitro* [17], was clearly up-regulated in KO cells (Figure 3H). Similarly, while claudin-3 showed similar expression in all three cell lines, claudin-4 was markedly increased in KO cells as compared to control and E-cad cells (Figure 3I and 3J). Previous studies have shown that ZO-3 and Claudin-4 are expressed in mammary epithelial cells or luminal subtypes of breast cancer such as MCF7 [18–20].

We also examined expression of specific transcriptional regulators implicated in EMT activation, but found no difference in levels of SNAIL, SLUG and TWIST between the three cell lines (Figure 3K).

Collectively, expression of E-cadherin seemed to induce some epithelial characteristics associated with adherens junctions, while depletion of ARHGEF11 mostly affected expression of tight junction components in MDA-MB-231 cells.

### Depletion of ARHGEF11 reduced cell proliferation and survival of cultured MDA-MB-231 cells

**Figure 4:**
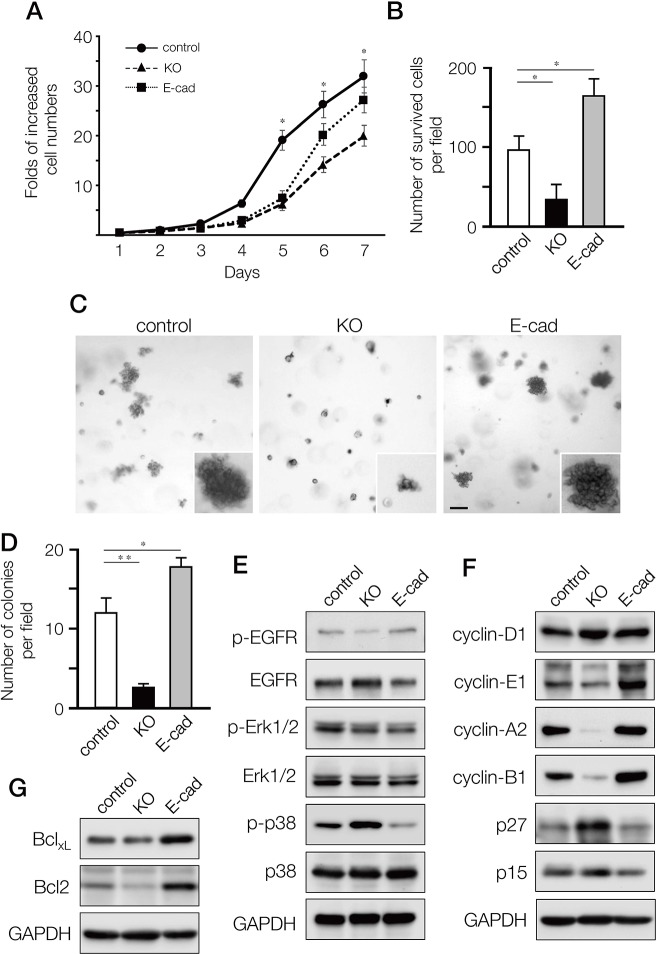
Effects on cell proliferation, growth and survival of MDA-MB-231 cells by depleting A11exon38(+) or forced expression of E-cadherin **(A)** Cell proliferation quantified daily for seven days, expressed as fold of increase with respect to cell number at day1. Data are mean±s.e.m. (n=3) ^*^P<0.05, unpaired two-tailed t-test. **(B)** The control, 231-KO and 231-Ecad cells were cultured under serum-free conditions for 2 days followed by culturing 3 days in the presence of 0.5% FBS. The number of surviving cells per field was counted. Data are mean±s.e.m. (n=3) ^*^P<0.05, unpaired two-tailed t-test. **(C, D)** Cells were cultured in the soft-agar plates for 18 days, then stained with crystal violet (C) and the number of survived colonies per field was quantified (D). **(E, F)** The lysates from control, KO and E-cad cells were immunoblotted with anti-p-EGFR, anti-EGFR, anti-p-Erk1/2, anti-Erk1/2, anti-p-p38, and anti-p38 (F), or anti-cyclinD1, anti-cyclinE1, anti-cyclinA2, anti-cyclinB1, anti-p27, and anti-p15. **(G)** The expression of anti-apoptotic Bcl family, Bcl_XL_ and Bcl2 was examined by western blotting.

We next assessed how depletion of ARHGEF11 affected the proliferation and/or survival of MDA-MB-231 cells, as compared to cells expressing E-cadherin or control cells. In proliferation assays, KO cells showed significantly reduced proliferation as compared to control cells, while proliferation of E-cad cells was somewhat attenuated (Figure 4A). In serum starvation assays, KO cells showed decreased survival, while E-cad cells showed increased survival, as compared to control cells (Figure 4B). Using soft-agar colony formation assays to investigate anchorage-independent growth, we found that KO cells formed significantly fewer colonies, while E-cad cells exhibited increased colony formation (Figure 4C and 4D). These results suggested that A11exon38(+) isoform might support not only migration and invasiveness but also proliferation and survival of invasive breast cancer cells, meanwhile, invasive breast cancer cells acquire greater ability for survival and proliferation by the re-expression of E-cadherin, as previously observed [21].

To define how the A11exon38(+) isoform affects proliferation and survival, we dissected key signaling cascades. While the EGFR-ERK signaling pathway showed minor differences between KO, E-cad, and control cells (Figure 4E), phosphorylation of p38 MAPK was increased in KO cells and decreased in E-cad cells compared to control cells. It has been shown that p38 MAPK could negatively regulate cell cycle progression both at the G1/S and the G2/M transitions by several mechanisms, including the downregulation of cyclins and upregulation of cyclin-dependent kinase (CDK) inhibitors [22]. We thus explored the expression of cyclins and CDK inhibitors. Cyclin-D1, which is a G1 cyclin, exhibited similar expression levels in those cells. On the other hand, cyclin A2 which regulates S phase progression and cyclin B1 that controls G2/M were significantly downregulated in KO cells and upregulated in E-cad cells, as cyclin-E1 that is G1/S cyclin (Figure 4F). Conversely, p27kip1, one of the CDK inhibitors, was upregulated in KO and downregulated in E-cad cells, while p15^INK4B^ CDK inhibitor, which function in G1, was equally expressed in the three cell lines.

In cancer cell survival, Bcl-2 family members play an important role. We found that anti-apoptotic factor Bcl-2 was decreased in KO cells and increased in E-cad cells, while Bcl-X_L_ expression was similar in KO and slightly upregulated in E-cad cells compared to control cells (Figure 4G).

Thus, depletion of ARHGEF11 in MDA-MB-231 cells induces a broad range of alterations in cell cycle and cell survival mediators, and these effects are partially mirrored by exogenous expression of E-cadherin.

### Expression of A11exon38(+) is necessary for tumor-forming ability of MDA-MB-231 cells

**Figure 5:**
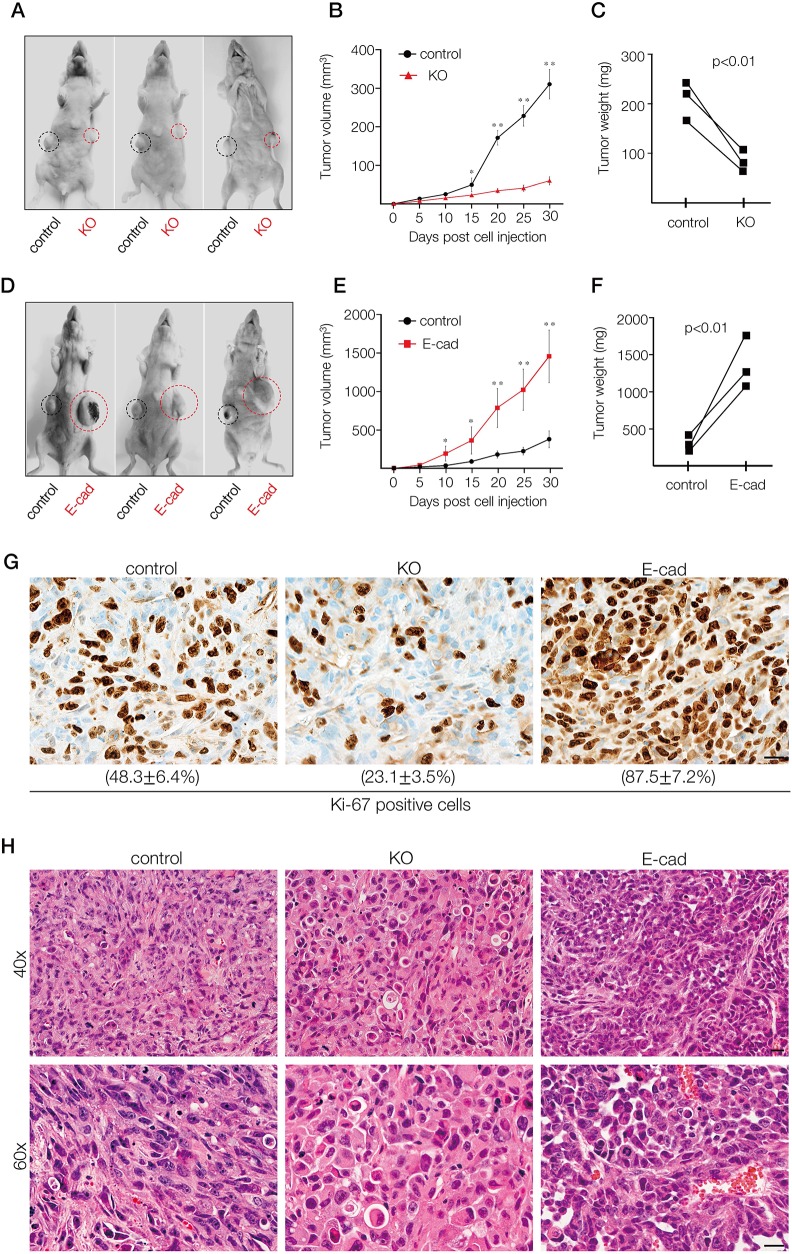
*In vivo* xenograft tumor formation of MDA-MB-231 cells depleted of A11exon38(+) or exogenously expressed E-cadherin **(A, D)** Control MDA-MB-231 cells (1 × 10^6^) were subcutaneously injected into the right flanks and the same number of KO cells (A) or E-cad cells (D) were injected into the opposite side of the same mice and followed for 30 days. Black and red circles indicate the tumors formed by control cells or manipulated cells. **(B, E)** Tumor volume was measured every 5 days until day 30 after injection. Data are mean±s.e.m. (n=3) ^*^P<0.05, ^**^P<0.01, unpaired two-tailed t-test. **(C, F)** Tumors were dissected at day 30, and weights were measured. **(G, H)** Representative images of tumor sections stained with Ki-67 antibody (G) or hematoxylin eosin (H) formed by control, KO or E-cad cells. The percentage of Ki-67 positive cells was quantified from three different experiments (G). Scale bars, 10μm.

We performed xenograft tumor assays to evaluate how the altered growth and invasion phenotypes induced by A11exon38(+) depletion or E-cadherin expression observed in culture affect tumor growth *in vivo*. The control cells and either KO or E-cad cells were subcutaneously injected into the mice and tumor growth was evaluated for 30 days (Figure 5A and 5D). The tumor growth was measured every 5 days (Figure 5B and 5E),and dissected tumors were weighed (Figure 5C and 5F). We observed that the xenograft tumor formation was significantly suppressed by the deletion of A11exon38 (+) (Figure 5A-5C), while exogenous expression of E-cadherin significantly increased the tumor growth *in vivo* (Figure 5D-5F).

To assess proliferation, sections of the dissected tumor were stained for Ki-67. As a result, ∼50% cells of control and ∼25% cells of KO samples were Ki-67 positive, while more than 80% cells of E-cad samples were positive for Ki-67 (Figure 5G). The histological analyses indicated that the tumor formed by E-cad cells appeared more densely packed than control or KO cells (Figure 5H). These data show that proliferation and/or survival of basal breast cancer cells *in vivo* could be suppressed by the depletion of ARHGEF11 isoform, suggesting that A11exon38(+) could be a target for therapy of invasive breast cancer.

## DISCUSSION

Here we demonstrate that the expression of the specific splice isoform of ARHGEF11 has critical impact in breast cancer cells. We previously identified ARHGEF11exon38(-), the isoform devoid of exon38, as a binding partner of the tight junction component ZO-1 [4, 5]. ARHGEF11exon38(-) is localized at tight junctions in a ZO-1-dependent manner in polarized epithelial cells, and is necessary for the function of PJAR. Here we show that invasive basal subtype breast cancer cells, which lack tight junctions and PJAR, express a distinct isoform of ARHGEF11 that contains exon38, ARHGEF11exon38(+). ARHGEF11exon38(+) exhibits the same RhoGEF and RGS activity as ARHGEF11exon38(-), but does not bind to ZO-1. Thus, the presence or absence of exon38 appears to specifically affect its ZO-1-binding ability, contributing in part to differential subcellular localization of the two isoforms. We further found that depletion of ARHGEF11 in MDA-MB-231 cells suppressed the highly motile and invasive characteristics, changed cell morphology from fibroblastic to epithelial-like pattern and reduced cell protrusion extension. In addition, growth and survival of MDA-MB-231 cells are suppressed both *in vitro* and *in vivo* by ARHGEF11exon38 depletion. Thus, inclusion of exon 38 in ARHGEF11 confers active, protumorigenic properties in breast cancer cells. The role of ARHGEF11-exon38(+) in invasion *in vivo* should be addressed in the near future.

EMT has been shown to play a critical role in progression of many cancer types, conferring invasiveness, metastasis, and resistance to cell death. Many studies of EMT in cancer have focused on transcriptional regulators, including members of the Snail and Twist families, on decreased expression of epithelial molecules such as E-cadherin, and increased expression of mesenchymal molecules such as N-cadherin. However, a recent study has shown how alternative splicing is an important modulator of EMT and invasive tumor progression in breast cells, finding that expression of Twist in mammary epithelial cells leads to altered splicing of more than 100 genes including ARHGEF11, which showed EMT-associated inclusion of exon 38 [9]. Importantly, the similar splicing changes were detected in samples from invasive ductal carcinoma patients for several genes, including ARHGEF11. Although a set of alternatively spliced genes were identified, functional differences between isoforms and the roles to tumor progression were addressed and elucidated only for a few genes.

In this study, we discovered a clear difference between two ARGEF11 isoforms expressed in non-invasive luminal subtype and invasive basal subtype cancer cells. The insertion of 32 amino acids coded by exon 38 of ARHGEF11 observed in invasive breast cancer cells abolished its interaction with ZO-1 and changed its subcellular localization from cell-cell junctions to cell edge and cytoplasm. It is possible that the depletion of ARHGEF11 results in reduced activation of RhoA at the cell edge, with consequent reductions in protrusion formation and cell migration/invasion. Combined with the previous studies that ARHGEF11 promotes dendrite formation in neuronal cells [23, 24], ARHGEF11 might play important roles in the formation of actin-based cell processes in various cell types.

The depletion of ARHGEF11 also changed cell morphology and upregulated the mRNA and protein expression of epithelial markers such as kertin8/18, although E-cadherin was not induced. Interestingly, while the expression of adherens junction components remained the same level with control cells, several tight junction components were upregulated in KO cells. Especially, claudin-4, of which low expression is associated with breast cancer stem cell phenotype and poor prognosis [25], is significantly increased. Since we had observed that decreased expression of ARHGEF11-exon38(+) increased luminal epithelial markers while increased E-cadherin expression did not, we assessed whether these changes promoted the ability of cells grown in Matrigel to re-polarize into luminal epithelial-like structures organized around a lumen. While we did not observe lumen development in either ARHGEF11-exon38(+)-decreased or E-cadherin-increased cells (data not shown), although this may not be surprising, as expression of luminal epithelial markers does not always accompany lumen formation, e.g., MCF7 or T47D cells, which both express luminal epithelial markers, do not form lumen when grown in Matrigel [26, 27]. These observations provide additional tools for dissection of the molecular mechanisms which regulate three-dimensional cellular architecture.

We also found that both proliferation and survival of MDA-MB-231 cells were suppressed by depleting ARHGEF11, not only *in vitro* but also *in vivo*. Our data demonstrated that ARHGEF11 depletion did not affect EGFR-Erk singling, but upregulated p38MAPK activity in MDA-MB-231 cells. Several studies suggested that p38MAPK regulates cell survival or cell death and functions as a tumor suppressor. The observation that cell survival factor Bcl-2 was decreased in KO cells may consistent with p38MAPK upregulation. Furthermore, expression of cyclin-E1, cyclin-A2 and cyclin-B1 were evidently decreased. These alterations could be due in part to the elevation of p27kip1 that suppresses cyclin-E, -A and –B in KO cells. It is also possible that the alteration of cell morphology was related to the reduction of cyclin expression from previous studies implicating cyclin expression in cytoskeletal morphology [28]. While the process by which ARHGEF11exon38(+) affects cell cycle mediators is still unknown, these results clearly connect the specific splice isoform of ARHGEF11 in growth of invasive breast cancer cells. It should be noted that we did not assess micrometastases in the lung or liver in our *in vivo* assay, because both control and manipulated cells were injected into the same mice. However, we did not see macroscopic differences for the lung or liver of mice injected with E-cadherin-expressing or ARHGEF11-KO cells (data not shown).

**Figure 6:**
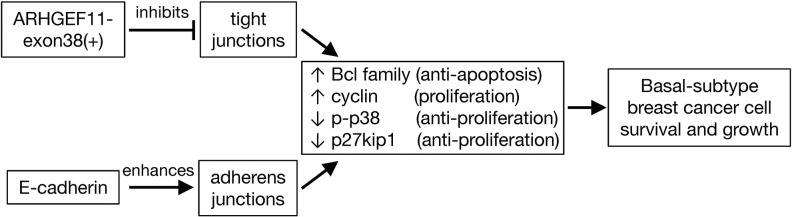
Model of effects of ARHGEF11-exon38(+) and E-cadherin expression in basal breast cancer cells In basal-subtype breast cancer cells, expression of ARHGEF1-exon38(+) inhibits tight junctions, while expression of E-cadherin promotes expression of adherens junctions. Promotion of cell-cell adhesion by either pathway in this cell type leads to increased activity of signaling pathways that block apoptosis and increase proliferation, driving the malignant phenotype.

E-cadherin is often downregulated or lost at the primary site in metastatic cancer, while metastases often re-express E-cadherin, a phenomenon which is attributed to EMT at the primary site, and reverse EMT, mesenchymal-epithelial transition, at the site of metastasis formation [29–31]. Our results that the forced expression of E-cadherin potentiated the growth and survival of MDA-MB-231 cells provide an experimental model in which these differential effects can be dissected, and comparison of these cells with parallel lines in which ARHGEF11exon38(+) is manipulated (Figure 6), provide insight into the differential effect of these two proteins on cancer cell phenotype.

In conclusion, our work suggested that defining the molecular basis of splicing and regulation of ARHGEF11 and other molecules will provide critical new insight into the invasive properties of breast cancer and provide new avenues for novel therapeutic interventions for treatment of patients in the future.

## MATERIALS AND METHODS

### Ethics statement

The care and use of all mice in this study were in accordance with the Guidelines for Proper Conduct of Animal Experiment (Science Council of Japan). All animal protocol was approved by the committee of the Care and Use of Laboratory Animals in Dokkyo Medical University (Permit Number: 06-517) and experimental work was performed in accordance with the ARRIVE guidelines. All efforts were made to minimize suffering including housing mice in a specific pathogen-free unit in which the light cycle was maintained at 12 h light/12 h dark and room temperature was 21±2°C. No more than 5 mice were housed in one cage and given food and water ad libitum. Mice were sacrificed by terminal anaesthesia with isoflurane followed by cervical dislocation.

### Cell culture

Human breast cancer cell lines BT-549, MDA-MB-231, MCF-7 and T47D as well as human embryonic kidney 293T cells were originated from American Type Culture Collection (ATCC, USA) and cultured in Dulbecco's modified Eagle's medium (DMEM) supplemented with 10% fetal calf serum (FCS). ZO-1 knockout mouse mammary epithelial cell line EpH4 was generated and cultured as previously described [3]. The cells generated in this study as described below were also maintained DMEM supplemented with 10% FCS. For three-dimensional cell cultures, cells grown as monolayers were trypsinized, suspended (3×10^5^ cells/ml) in DMEM supplemented with 2% FCS and 2% (vol/vol) matrigel, then plated on polymerized matrigel.

### Antibodies and reagents

The specific antibody against ARHGEF11exon38(+) isoform was raised using a peptide coded by exon38, SKVVPALPESGQSEP, as an antigen. Mouse mAbs against RhoA, Rac1, CDC42, E-cadherin, α-catenin, β-catenin, and p27 were obtained from BD Biosciences; rabbit Abs against p-EGFR, EGFR, p-Erk1/2, Erk1/2, p-p38, p38, cyclin-D1, cyclin-E1, cyclin-A2, cyclin-B1, ARHGEF2, keratin8/18, and vimentin were from Cell signaling; rabbit pAb against N-cadherin was from EMD; rabbit Abs against ARHGEF18, G_α13_, p15, and Ki67 were from Genetex; rabbit Abs against ZO-1, ZO-2, ZO-3, occludin, claudin-3, and claudin-4 were from Invitrogen; mouse mAbs against ARHGEF11, Bcl-2, and Bcl-xL were from Santa Cruz Biotech.; mouse mAb against vinculin was from Sigma; mouse mAbs against GAPDH, GFP, and Myc were from Wako; Alexa Fluor 488/594-conjugated anti-mouse or anti-rabbit IgG, and Alexa Fluor 594-conjugated phalloidin were from Invitrogen; HRP-conjugated anti-mouse or anti-rabbit IgG were from Santa Cruz Biotech.

### Generation of ARHGEF11-knockout MDA-MB-231 cells by CRISPR/Cas-9

MDA-MB-231 cells depleted of ARHGEF11exon38(+) were generated by CRISPR/Cas-9 double-nickase system. The two pairs of single-guide RNAs (sgRNAs) targeting human ARHGEF11 exon 1 were designed; #1 (5′-CACCGTTCCAGAATCCTGTAGCTT-3′; 5′-AAACAAGCTACAGGATTCTGGA ACC) and #2 (5′-CACCGGGTTACCCCAGAGTATAGAC-3′; 5′-AAACGTCTATACTCTGGGGTA ACCC-3′). The sgRNA pairs were cloned into a pSpCas9n(BB)-2A-Puro (PX462) vector (Addgene plasmid 48141). These vectors were co-transfected into MDA-MB-231 cells using Lipofectamine 2000 transfection reagent (Life Technologies, Inc.), according to the manufacturer's instructions. After 48 hours, the cells were cultured in the presence of 10 μg/ml puromycin for 10 days, then surviving cell colonies were expanded and screened for ARHGEF11exon38(+) expression by western blotting, as described below. The genomic DNA from clones with a significant ARHGEF11exon38(+) reduction was isolated and the DNA fragments for the sgRNA target regions were amplified by PCR and sequenced directly to verify the deletion mutation regions. To rescue ARHGEF11 expression, ARHGEF11exon38(-) or ARHGEF11exon38(+) subcloned into pCAGGS-myc vector was transfected into KO cells using Lipofectamine 2000.

### Establishment of MDA-MB-231 cells stably expressing E-cadherin

The coding region of mouse E-cadherin was subcloned into pCAGGS-neo vector and transfected into MDA-MB-231 cells using Lipofectamine 2000. After 48 hours, the cells were cultured in the presence of 1 mg/ml G418 for 14 days. The E-cadherin positive cells were screened by western blotting and immunofluorescence.

### Migration and invasion assays

The migration and invasion capacity of cells were evaluated in 12-well chambers with 8 μm pore transwell filter inserts or biocoat matrigel invasion chamber (Corning) according to the manufacturer's instructions. Briefly, 2 × 10^5^ cells were plated into the upper chamber in 300 μL of culture medium. The lower chamber was filled with 350 μL of medium containing 5% FCS. After culture for 10 hour, cells were fixed with methanol and stained with 0.5% crystal violet for 10 min. Cells on the upper side of the filter were removed with a cotton swab, and cells on the lower side of the filter were visualized and counted. Each experiment was repeated three times in duplicate.

### Cell proliferation and survival assay

Cell proliferation was measured by counting cell number every day after seeding for 7 days. Cell survival ability was assessed by culturing cells under serum-free conditions for 2 days followed by culture for 3 days in the presence of 0.5% FBS. The surviving colonies comprised of more than 50 individual cells were counted.

### The soft agar colony formation assay

Cells (2 x10^4^) were suspended in 0.36% agarose and layered on top of prepared 0.75% bottom agar plates in DMEM containing 10% FBS. The agar plates were incubated at 37°C and cultured for 2 weeks, followed by staining with 0.005% crystal violet. Then the number of colonies were counted from three independent dishes of each sample.

### *In vivo* tumor formation assay

Tumor formation ability *in vivo* was examined by injecting 1 × 10^6^ cells subcutaneously into the bilateral flanks of Balb/c female athymic nude mice (CLEA Japan, Inc.) at 6 weeks of age. Tumor growth was monitored every 5 days. The tumors were harvested after 30 days and their weights were measured. A portion of tumors was embedded in paraffin, sectioned, and stained with Hematoxylin/Eosin for histology or with Ki67 for clarifying cell proliferation. All experiments involving animals were performed according to protocols approved by the committee of the Care and Use of Laboratory Animals in Dokkyo Medical University.

### RNA isolation and RT-PCR analysis

Total RNA was isolated using the RNeasy Mini Kit (Qiagen) and then reverse-transcribed using ReverTra Ace qPCR RT Master Mix (TOYOBO), according to the manufacturer's instructions. Quantitative RT-PCR was carried out on a StepOne Real Time PCR System (Applied Biosystems) using FastStart Universal SYBR Green Master (Roche Applied Science). mRNA levels were normalized to GAPDH and are shown as the relative fold expression compared to respective control. All the primers used for quantitative RT-PCR are listed in Supplementary Table 1. To detect ARHGEF11 isoforms including or skipping exon 38, RT-PCR was performed with primers flanking exon 38; forward (5′-CCAAGGACCAGAAATTCTGG-3′) and reverse (5′-CAGCTCTCTGTGGGCCAGCTCCA-3′).

### SDS-PAGE and western blot

One-dimensional SDS-PAGE was performed with standard protocol and the separated proteins were transferred from the gels onto PVDF membranes. The membranes were soaked in 5% skim milk and incubated with the primary antibodies. After being washed with TBS containing 0.2% Tween-20 (TBS-T), the membranes were incubated with HRP-conjugated secondary antibodies for rabbit or mouse IgG (Santa Cruz Biotech.). They were then washed with TBS-T followed by incubation with Clarity Western ECL substrate (Bio-Rad). The signal was captured using the ChemiDoc system and analyzed by Quantity One software (Bio-Rad).

### Immunofluorescence staining

Cells cultured on glass coverslips were fixed with 3% formalin or with 100% methanol. The fixed cells were incubated with appropriate primary antibodies, followed by incubating with Alexa fluor labeled secondary antibodies. Images were taken using confocal microscope equipped with 63x oil lens (Zeiss LSM 710) and processed by ZEN software (Zeiss).

### *In vitro* binding assay and immunoprecipitation

The GST fusion protein of ZO-1 fragments (aa 1520-1745) was expressed in *E. coli* JM109 in the presence 0.1 mM IPTG. The *E. coli* were then lysed by brief sonication in buffer A (20 mM Tris-HCl, 150 mM NaCl, 1 mM EDTA, 10% Glycerol, 1% NP-40, 1 mM PMSF, pH 7.4). The fusion proteins (∼10 μg) were purified on glutathione-Sepharose 4B beads (GE Healthcare), followed by incubation with lysates of 293T cells transfected with pCAGGS-myc- ARHGEF11exon38(-) or pCAGGS-myc-ARHGEF11exon38(+) expression vectors. The beads were washed with buffer A, then treated with elution buffer (50 mM Tris-HCl, 25 mM glutathione, pH 8.8). For immunoprecipitation, 293T cells were co-transfected with pCAG-GFP-ZO-1 and pCAGGS-myc- ARHGEF11exon38(-) or pCAGGS-myc-ARHGEF11exon38(+). The cell lysates were incubated with anti-myc antibody-conjugated agarose beads (Wako) for 1 hr. After wash with buffer A, immuno-complex was eluted with myc peptides. The lysates and eluates were analyzed by western blotting.

### Activation assays for RhoA, Rac1, CDC42 and G_α13_

The activity of RhoA, Rac1 and CDC42 was measured using RhoA/Rac1/CDC42 Activation Biochem kit (Cytoskeleton, Inc.), according to the manufacturer's instructions. Briefly, cells were lysed with lysis buffer (50mM Tris-HCl, pH 7.5, 500mM NaCl, 2% NP40, 10mM MgCl_2_) containing protease inhibitor cocktail at 4°C for 30 min. Lysates were cleared by centrifugation at 15,000*g* at 4°C for 15 min. Supernatants were incubated with 30 μg of Rhotekin-RBD beads (for RhoA) or PAK-PBD beads (for Rac1 and CDC42) for 1 hour at 4°C. Beads were washed three times in wash buffer (25mM Tris-HCl, pH 7.5, 40mM NaCl, 30mM MgCl_2_) and resuspended in Laemmli buffer. To measure G_α13_ activity, GST-TPR (kindly provided by Dr. M. Neghishi) bound to glutathione-Sepharose beads was incubated with cell lysates prepared in lysis buffer (20mM Hepes, pH 8.0, 2mM MgCl_2_, 1mM EDTA, 0.1% Triton X-100) for 1 hour at 4°C. After the beads were washed with the lysis buffer, the bound proteins were eluted in Laemmli buffer. The lysates and eluted samples were analyzed by immunoblotting.

### Statistical analysis

For statistical analysis of the data, experiments were repeated three times unless stated otherwise and data are presented as mean ± SEM. Student´s *t*-test was used for pairwise comparison of the data and significance calculations. *P* values < 0.05 were considered statistically significant.

## SUPPLEMENTARY MATERIALS TABLES




