# Supplementary material for: The exon 38-containing ARHGEF11 splice isoform is differentially expressed and is required for migration and growth in invasive breast cancer cells
Source: Oncotarget. 2017 Sep 18;8(54):92157–70. doi: 10.18632/oncotarget.20985 (PMC5696171; doi:10.18632/oncotarget.20985)
Supplement: Supplementary file 1 [file oncotarget-08-92157-s001.pdf]

The exon 38-containing ARHGEF11 splice isoform is differentially expressed and is required for migration and growth in invasive breast cancer cells

SUPPLEMENTARY MATERIALS

Supplementary Table 1: qPCR primer sequences

See Supplemetary File 1
